# Protective Effects of Chaya against Mitochondrial and Synaptic Toxicities in the Type 2 Diabetes Mouse Model TallyHO

**DOI:** 10.3390/cells11040744

**Published:** 2022-02-21

**Authors:** Bhagavathi Ramasubramanian, Cameron Griffith, Madison Hanson, Lloyd E. Bunquin, Arubala P. Reddy, Vijay Hegde, P. Hemachandra Reddy

**Affiliations:** 1 Department of Internal Medicine, Texas Tech University Health Sciences Center, 3601 4th Street, Lubbock, TX 79430, USA; b.ramasubramanian@ttuhsc.edu (B.R.); Cameron.Griffith@ttu.edu (C.G.); madisonhanson08@gmail.com (M.H.); lloyd.e.bunquin@ttuhsc.edu (L.E.B.); 2 Department of Sociology, Anthropology, and Social Work, Texas Tech University, Lubbock, TX 79409, USA; 3 Nutritional Sciences Department, College of Human Sciences, Texas Tech University, 1301 Akron Ave, Lubbock, TX 79409, USA; arubala.reddy@ttu.edu (A.P.R.); vijay.hegde@ttu.edu (V.H.); 4 Department of Pharmacology and Neuroscience, Texas Tech University Health Sciences Center, Lubbock, TX 79430, USA; 5 Department of Neurology, Texas Tech University Health Sciences Center, Lubbock, TX 79430, USA; 6 Department of Public Health, Graduate School of Biomedical Sciences, Texas Tech University Health Sciences Center, Lubbock, TX 79430, USA; 7 Department of Speech, Language, and Hearing Sciences, Texas Tech University Health Sciences Center, Lubbock, TX 79430, USA

**Keywords:** chaya chow, Type 2 diabetes, TallyHO mice, mitochondrial biogenesis, synaptic proteins

## Abstract

The purpose of our study is to determine the protective effects of the chaya leaf against mitochondrial abnormalities and synaptic damage in the Type 2 diabetes (T2D) mouse model, TallyHO (TH). The TH mouse is a naturally occurring polygenic mouse model of diabetes that mimics many characteristics of human Type 2 diabetes. Only male TH mice develop hyperglycemia and moderate obesity. Female mice display moderate obesity but do not manifest overt diabetes. In this study, we evaluated three groups of mice over a period of 11 weeks: (1) the experimental group of TH diabetic mice fed with chaya chow; (2) a diabetic control group of TH diabetic mice fed with regular chow; and (3) a non-diabetic control group of SWR/J mice fed with regular chow. Body mass and fasting blood glucose were assessed weekly. Brain and other peripheral tissues were collected. Using qRT-PCR and immunoblotting analyses, we measured the mRNA abundance and protein levels of mitochondrial biogenesis, mitochondrial dynamics, autophagy/mitophagy, and synaptic genes. Using immunofluorescence analysis, we measured the regional immunoreactivities of mitochondrial and synaptic proteins. Using biochemical methods, we assessed mitochondrial function. We found increased body mass and fasting glucose levels in the TH diabetic mice relative to the non-diabetic control SWRJ mice. In chaya chow-fed TH diabetic mice, we found significantly reduced body mass and fasting glucose levels. Mitochondrial fission genes were increased and fusion, biogenesis, autophagy/mitophagy, and synaptic genes were reduced in the TH mice; however, in the chaya chow-fed TH diabetic mice, mitochondrial fission genes were reduced and fusion, biogenesis, autophagy/mitophagy, and synaptic genes were increased. Mitochondrial function was defective in the diabetic TH mice; however, it was rescued in the chaya chow-fed TH mice. These observations strongly suggest that chaya chow reduces the diabetic properties, mitochondrial abnormalities, and synaptic pathology in diabetic, TH male mice. Our data strongly indicates that chaya can be used as natural supplemental diet for prediabetic and diabetic subjects and individuals with metabolic disorders.

## 1. Introduction

Type 2 diabetes mellitus is a chronic disorder due to insulin resistance resulting in the dysregulation of glucose homeostasis, hyperglycemia, and vascular complications [1]. The prevalence of diabetes and metabolic disease is rapidly increasing worldwide and is becoming a major health issue with related personal, social, and economic burdens. The International Diabetes Federation recently reported 463 million adults currently living with diabetes. If no action is taken to address this pandemic, it is estimated that 578 million people will have diabetes by 2030, which will further increase to 700 million by 2045. The annual global health expenditure on diabetes has been estimated at USD 760 billion. Based on this, the estimated direct costs to manage diabetes will be USD 825 billion by 2030 and USD 845 billion by 2045 [2].

The high prevalence, variable pathogenesis, progressive process, and complications of diabetes highlight the need for effective treatments. Drugs derived from natural products such as chaya are often more suitable than synthetic drugs because of their diversity and minimal side effects [3]. Assessments of plant-based therapeutics have shown that some natural compounds can directly enhance insulin secretion, prevent pancreatic beta cell apoptosis, and modulate pancreatic beta cell differentiation and proliferation. Hence, natural compounds can be explored as sources of novel pharmaceuticals [4].

The plant *Cnidoscolus chayamansa*, commonly known as “chaya” or tree spinach, is native to the Maya regions of Central America [5]. Chayamansa is the most widely cultivated variety of the *Cnidoscolus aconitifolius* plant (ssp *aconitifolius Breckon*) due to the fact that it lacks the stinging hairs present in other varieties that can irritate the skin during the harvesting process. When cooked and eaten as a vegetable food item, chaya provides high percentages of vitamin C, beta carotene, protein, calcium, phosphorous, iron, thiamin, riboflavin, and niacin [6].

Many indigenous communities of the Yucatán Peninsula of Mexico and Belize have reported that they use chaya for various medicinal purposes [7,8]. Some traditional uses include the treatment of kidney stones, hemorrhoids, and eye problems, as well as improving blood circulation, decongesting lungs, stimulating lactation, improving memory and brain function, lowering cholesterol, and treating diabetes [9]. Of these, the claims of the hypoglycemic and antidiabetic properties of chaya have been shown to have merit in a few preliminary laboratory experiments [6,10,11,12]. However, more studies will definitely be necessary to determine the effective dosage needed to lower blood sugar levels, the mechanism of the hypoglycemic activity and the active hypoglycemic component present in the leaves of chaya plant [13,14]. The studies thus far reported were conducted in STZ-induced Type 1 diabetes rodent models. For the first time, we report here the effects of chaya in the T2D mouse model TallyHO.

The TH mouse is a naturally occurring polygenic mouse model of diabetes that mimics many characteristics of human non-insulin-dependent Type 2 diabetes mellitus [15]. Male TH mice develop hyperglycemia, hyperinsulinemia, hyperlipidemia, moderate obesity, and enlargement of the islets of Langerhans at the age of 16 weeks. Female mice display moderate hyperinsulinemia and obesity, but do not manifest overt diabetes [16,17]. Recent genetic studies identified many SNPs and environmental factors that contribute to the development of diabetes in TH mice [18,19]. These mice showed increased body mass as compared to C57BL/6J mice [20].

Studies have demonstrated that T2D also leads to the cognitive dysfunction characteristic of Alzheimer’s disease (AD) in another diabetic mouse model, *db*/*db*. Individuals with T2D are at increased risk of dementia compared with those without diabetes [21,22]. Mitochondrial dysfunction, including impaired mitochondrial biogenesis, was reported as an important contributing factor to insulin resistance in T2D [21,22]. Our group previously reported mitochondrial alterations in TH mice relative to non-diabetic control mice from liver and pancreatic tissues [17]. Since our aim is to understand the potential link between AD and T2D, in this study, we focus on assessing the mitochondrial and synaptic changes in the brain tissue of TallyHO mice fed with chaya chow relative to TallyHo mice fed with regular chow. We compare the TH groups to the parallel control groups of nondiabetic SWR/J mice. To our knowledge, this is the first study to examine mitochondrial and synaptic activities and mitochondrial function in the brain tissues of TH diabetic mice.

## 2. Materials and Methods

### 2.1. Animals

The diabetic mice used in this study were the inbred TallyHo/JngJ strain (TH) and the non-diabetic mice were the SWR/J strain. Both strains were obtained from the Jackson Laboratory. All the animals were housed in clean polypropylene cages and fed a standard chow diet ad libitum, with free access to water under controlled temperature and humidity, and with a 12-h light/dark cycle. All the experiments were performed according to the guidelines for use and care of laboratory animals and were approved by the Institutional Animal Care and Use Committee (IACUC) of the Texas Tech University Health Sciences Center, Lubbock, TX, USA. The body mass of the mice was measured every week for a period of 11 weeks. An intraperitoneal glucose tolerance test (IPGTT) was performed on all the animals at three different time points, beginning at 10 months of age. Briefly, the mice fasted overnight and their baseline blood glucose levels were measured in blood using a portable glucometer (AlphaTRAK 2 blood glucose monitoring system kit) via tail nick. Fasting blood glucose was also determined every week, beginning at 10 months of age, for a total of 11 weeks.

### 2.2. Chaya 

Chaya (*Cnidoscolus chayamansa*) contains a modicum of naturally occurring cyanogenic glycocide, a toxin found in various plants. Thus, chaya must be boiled or heat-dried in order to bring the level of toxin to within the range acceptable for human consumption. Our approach to the introduction of chaya leaf meal to the diet of laboratory mice was modeled after previous experiments by Sarmiento-Franco et al. [23], and lab tests in Belize have demonstrated that the drying protocol for the leaves used in our experiments yielded no residual cyanide (J. Cho, personal communication to C. Griffith, 2 November 2018).

Chaya leaves were harvested from an organic farm in San Felipe, Belize. Only healthy, green leaves of the *estrella* variety were selected, and the stems were removed. The chaya leaves were then dried on stainless steel drying trays at 165 degrees Fahrenheit for 5 h in a Crawford 1000-watt electric commercial food dehydrator. The dried leaves were then ground to a fine powder using a Vitamix 5300 Professional Grade blender. The chaya leaf meal powder was then heat-sealed in plastic bags and shipped to Dr. Jamie Lecker of Bio-Serv, Inc., where it was incorporated into Teklad 2018, an industry standard grain-based rodent diet. The resultant product, ‘chaya chow,’ was infused with 25% chaya. The chaya chow was then gamma irradiated to mitigate any pathogens.

### 2.3. Treatment of Animals

We selected fully diabetic 10-month-old male TH mice because these mice were readily available in our facility and our long-term goal is to study cognition in aging TH mice to understand the link between T2D and Alzheimer’s disease. The mice were divided into three groups fed with either regular chow feed or chaya chow feed for a period of 11 weeks and then compared to their age-matched, male, non-diabetic SWR/J mice, as shown below:

Group 1—TH diabetic mice fed with chaya chow (diabetic experimental group)
Group 2—TH diabetic mice fed with regular chow (diabetic control group)
Group 3—SWR/J mice fed with regular chow (non-diabetic control group)

**Figure 1:**
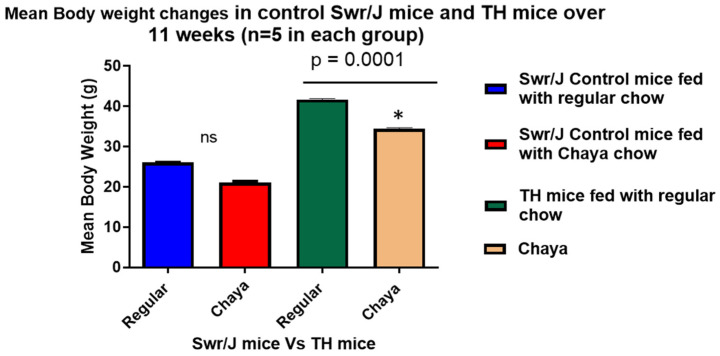
A comparison of the changes in body mass in the TH mice fed with chaya and TH mice fed with regular chow over a period of 11 weeks in relation to the SWR/J control mice with a statistical significance between the two TH mice groups (*n* = 5, *p* = 0.0001). * *p* = 0.01.

**Figure 2:**
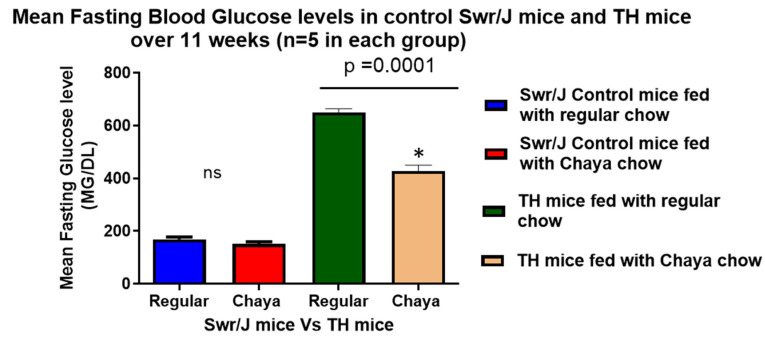
A comparison of the blood glucose levels in the TH mice fed with chaya and TH mice fed with regular chow over a period of 11 weeks (*p* < 0.005) in relation to the Swr/J control mice with a statistical significance between the two TH mice groups (*n* = 5, *p* = 0.0001). * *p* = 0.01.

Since our initital studies did not indicate any change between chaya-fed SWR/J mice and regular chow-fed SWR/J mice in relation to body mass and blood glucose levels (Figure 1 and Figure 2), we used only regular chow-fed SWR/J mice as the non-diabetic control in the study. At the end of 11 weeks, fasting blood glucose was assessed and the animals were euthanized by cervical dislocation. Blood samples and tissues such as liver, pancreas, brain, and skeletal muscle were collected.

### 2.4. Quantitative Real-Time PCR (qRT-PCR)

Gene expression analysis was performed using qRT-PCR according to our lab-published methods [17,24]. The oligonucleotide primers were designed using primer express software (Applied Biosystems, Carlsbad, CA, USA) for the housekeeping genes β-actin and GAPDH, mitochondrial structural genes, fission genes (Drp1 and Fis1), fusion genes (MFN1, MFN2, and Opa1), biogenesis genes (PGC1 α, TFAM, Nrf1, and Nrf2), synaptic genes (synaptophysin and PSD95), and autophagy/mitophagy genes (ATG5, PINK1, and TERT), as described in our previous studies [17,24]. The primer sequence for the above-mentioned genes is listed in Table 1. To determine the statistical significance of mRNA expression, the experiments were repeated three times and the CT value differences between the chaya-fed TH mice and regular chow-fed TH mice were used in relation to β-actin normalization.

### 2.5. Western Blotting Analysis

To determine the impact of hyperglycemia and chaya treatment on the protein levels of the mitochondrial dynamics and biogenesis genes, we performed immunoblotting analyses of protein lysates from the brain tissues of experimental groups as described in our previous studies [17,24]. Details of antibody dilutions and other information are given in Table 2. Densitometric analysis to quantify the bands was done using Image J software as per NIH guidelines.

### 2.6. Immunostaining Analysis

To determine the immunoreactivities of the mitochondrial dynamics, biogenesis, synaptic, and autophagy/mitophagy proteins, immunofluorescence analysis was performed on brain sections from non-diabetic animals, diabetic animals that were fed with chaya, and control diabetic animals that were fed with regular chow. Details of the antibody dilutions are given in Table 3. Briefly, the frozen tissue sections were fixed in freshly prepared 4% paraformaldehyde in PBS for 15 min, and then washed with PBS and permeabilized with 0.1% Triton-X100 in PBS as described previously [17,24]. They were blocked with 10% goat serum blocking solution (Life technologies) for 1 h at room temperature. All sections were incubated overnight with the antibodies and dilutions described in Table 3. After incubation, the sections were washed three times with PBS, for 10 min each time. The sections were incubated with a secondary antibody conjugated with Fluor 488 (Invitrogen) for 1 h at room temperature. The sections were then washed three times with PBS and mounted on slides. Photographs were taken with an Olympus IX 83 microscope. To quantify the immunoreactivities of antibodies, 10–15 photographs were taken at ×40 magnifications and statistical significance was assessed using cellSens software analysis.

### 2.7. Mitochondrial Functional Assays

Using cortical lysates from chaya chow-fed TH mice and the corresponding age-matched, non-diabetic control mice, we assessed mitochondrial function by measuring H_2_O_2_, lipid peroxidation, and ATP levels.

H_2_O_2_ production levels were measured using an Amplex^®^ Red H_2_O_2_ assay kit (Molecular Probes, Eugene, OR, USA). Briefly, the reaction mixture contained tissue lysate, Amplex Red reagents, horseradish peroxidase, and a reaction buffer. The mixture was incubated at room temperature for 30 min, followed by spectrophotometer readings of absorbance at 570 nm. H_2_O_2_ production was determined using a standard curve equation expressed in nmol/μL of the lysate.

4-hydroxy-nonenol (4-HNE) levels were measured using a lipid peroxidation assay kit (Abcam, Boston, MA, USA). Briefly, the selected wells of a 96-well plate were coated overnight at 4 °C with 4-HNE conjugate. After washing three times, the samples and standards were added to the coated plate, which was then incubated for 1 h at room temperature. The samples and standards were incubated with a secondary antibody conjugated with peroxidase for 1 h at room temperature, followed by incubation with an enzyme substrate. Optical density was measured at 450 nm to quantify the level of HNE.

Mitochondrial ATP levels were measured using an ATP assay kit (Abcam, Boston, MA, USA). The ATP levels were measured in isolated mitochondria as per the instructions in the kit. The absorbance was measured at 570 nm and the ATP levels were determined using a standard curve equation.

### 2.8. Statistical Analysis

Values were expressed as mean ± standard deviation. The data were analyzed using *t*-tests and non-parametric tests between the treatment groups. Values with *p* < 0.05 (two-tailed) were considered statistically significant. Statistical analysis was performed using GraphPad Prism for Windows, version 9 (GraphPad Software, GraphPad, San Diego, CA, USA).

## 3. Results

We previously showed that the body mass of TH male and female mice were significantly higher than the age- and sex-matched SWR/J control mice [17,25]. In this study, we found that the body mass of the chaya chow-fed male TH mice was significantly lower than the body mass of their age-matched regular chow-fed male TH mice (*p* = 0.0001), suggesting that chaya treatment helped lower the body mass of these mice. However, we did not see any change in the body mass between chaya chow-fed and regular chow-fed mice in the control group (Figure 1).

### 3.1. Hyperglycemia

Our previous studies indicated that only male TH mice developed hyperglycemia [17,24]. In this study, we found that the chaya chow-fed male TH mice showed a significant reduction in glucose levels over a period of 11 weeks of treatment (*p* = 0.0001) compared to the regular chow-fed TH mice. However, we did not see any change in the blood glucose levels between chaya chow-fed and regular chow-fed mice in the control group (Figure 2). These findings demonstrate the hypoglycemic properties of chaya in diabetic mice, similar to the previously reported experiments with chaya in streptozotocin-induced rats [10,11,12,13,14].

We did not observe any phenotypic and/or visual toxic effects due to chaya treatment in the SWR/J control mice compared to the regular chow-fed Swr/J mice during the study, and upon harvesting, tissues such as the brain, liver, pancreas, and kidney appeared to be intact.

### 3.2. mRNA Levels of Mitochondrial Dynamics, Biogenesis, Autophagy, Mitophagy, and Synaptic Genes

To determine the effect of chaya treatment in diabetic TH mice, we isolated total RNA from the brain tissues of all three groups of mice (the non-diabetic control mice, the diabetic TH mice fed with regular chow, and the TH mice fed with chaya chow). The mRNA levels of mitochondrial dynamics genes (fission Drp1 and Fis1, and fusion Mfn1, Mfn2, and Opa1), mitochondrial biogenesis genes (PGC1α, NRF1, NRF2, and TFAM), autophagy (ATG5 and PINK1), mitophagy (TERT), and synaptic genes (PSD95 and synaptophysin) in the control and TH mice fed with regular or chaya chow were measured using Sybr-Green chemistry-based quantitative real time RT-PCR.

Mitochondrial dynamics genes: As shown in Table 4, in TH mice fed with regular chow, the mRNA levels of mitochondrial fission genes were significantly increased (Drp1 by 5.7-fold, *p* = 0.0015 and Fis1 by 6.7-fold, *p* = 0.0005) compared to the control SWRJ mice. In contrast, the mRNA expression levels of mitochondrial fusion genes were significantly decreased (Mfn1 by 4.1-fold, *p =* 0.0001; Mfn2 by 4.5-fold, *p =* 0.0001; and Opa1 by 3.1, *p =* 0.0001) in TH mice fed with regular chow relative to the control mice. This indicates the presence of abnormal mitochondrial dynamics in TH mice. The TH mice fed with chaya chow showed a significant reduction in Drp1 (*p* = 0.005). In these chaya chow-fed TH mice, a statistically significant reduction of Fis1 (*p* = 0.0045) was also seen. The fusion genes were also increased in the chaya-fed TH mice relative to the regular chow-fed TH mice at significant levels (Mfn1 *p* = 0.0004, Mfn2 *p* = 0.0080, and Opa1 *p* = 0.0047) (Table 4). These observations indicate that chaya reduced fission activity and enhanced fusion machinery in TH mice.

Mitochondrial biogenesis genes. The mRNA levels of mitochondrial biogenesis genes were significantly reduced (PGC1α by 2-fold, *p* = 0.0001; NRF1 0.5, *p* = 0.0001; NRF2 0.1, *p* = 0.0001; and TFAM by 1.1, *p* = 0.0001) in TH mice relative to the control SWRJ mice (Table 4). However, in chaya chow-fed TH mice, the mRNA levels of mitochondrial biogenesis PGC1α, Nrf1, and Nrf2 showed a trend towards increasing levels, although they were not statistically significant. The mRNA levels of TFAM showed significantly increased levels in chaya chow-treated TH mice (TFAM *p* = 0.0240). These observations strongly suggest that chaya increases mitochondrial biogenesis activity in TH mice.

Autophagy genes. In TH mice relative to control mice, the mRNA levels of autophagy genes were significantly reduced (ATG5 by 3.1-fold, *p* = 0.0002 and PINK1 by 4.6-fold, *p* = 0.0315) (Table 4). On the contrary, in chaya chow-fed TH mice relative to regular chow-fed TH mice, the mRNA levels of autophagy genes were increased (ATG5 by 3.9-fold, *p* = 0.0352 and PINK1 by 8.7-fold, *p* = 0.0052) (Table 4). These observations suggest that chaya increases autophagy activity in TH mice.

Mitophagy genes. As shown in Table 4, in TH mice, the mRNA levels of the mitophagy gene TERT were significantly reduced by 4.1-fold, *p* = 0.0001, relative to control mice, indicating that mitophagy activities are reduced in TH mice. However, chaya chow-fed TH mice showed a significant increase in TERT levels by 3.4-fold (*p* = 0.0381), suggesting that chaya treatment is capable of enhancing mitophagy activity in TH mice.

Synaptic genes. As shown in Table 4, the mRNA levels of synaptic genes were significantly reduced (synaptophysin by 4.0-fold, *p* = 0.0001 and PSD95 by 3.7-fold, *p* = 0.0001) in TH mice relative to the control SWRJ mice. However, in chaya chow-fed TH mice relative to regular chow-fed TH mice, the mRNA levels of synaptic genes were increased (synaptophysin by 2.5-fold, *p* = 0.0309 and PSD95 by 3.4-fold, *p* = 0.0164). These observations indicate that chaya treatment may lead to an increase in the expression of genes involved in synapse formation.

### 3.3. Immunoblotting Analysis

In order to study the effect of chaya on TH mice at the protein level, we conducted immunoblot analyses of mitochondrial dynamics, mitochondrial biogenesis, autophagy, mitophagy, and synaptic proteins using protein lysates from hippocampal tissues.

**Figure 3:**
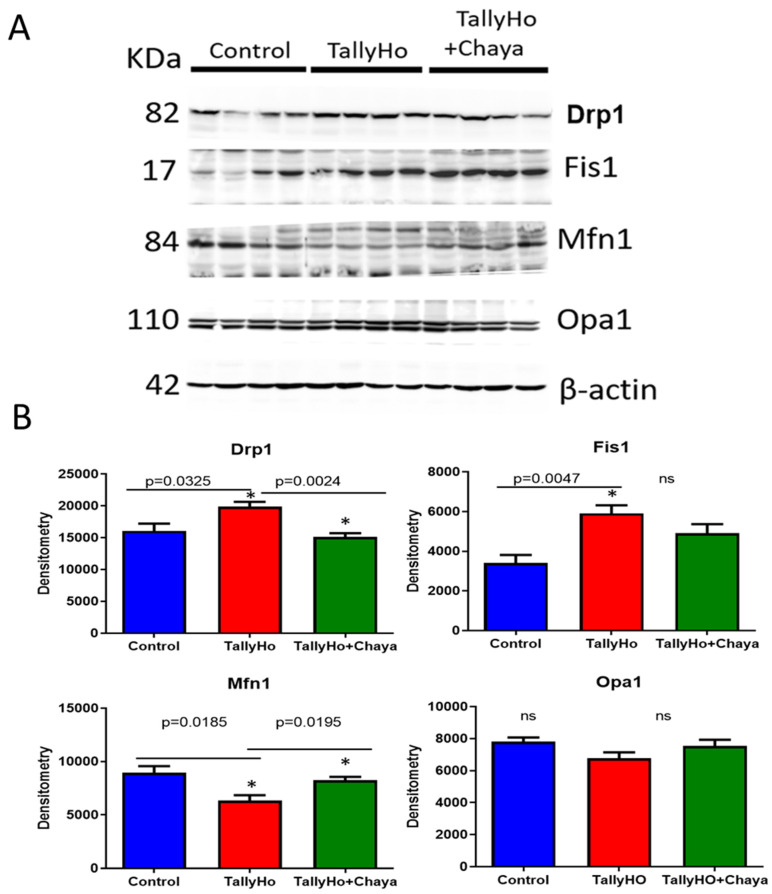
Immunoblotting analysis of mitochondrial dynamics, fission, and fusion proteins using protein lysates obtained from the post-mortem brains of 14-month-old non-diabetic control mice, TH mice fed with chaya, and TH mice fed with regular chow (for each of the three groups, *n* = 4). (**A**) Immunoblots for the control, TH, and TH + chaya mice. (**B**) Quantitative densitometry analysis for the mitochondrial fission proteins Drp1 and Fis1, which were significantly increased in the TH mice compared to the control mice (Drp1 *p* = 0.0325 and Fis1 *p* = 0.0047), but the Drp1 levels were significantly reduced in TH + chaya mice at levels comparable to the control mice (Drp1 *p* = 0.0024). Fis1 did not show any significant change in expression in the chaya-fed TH mice. The quantitative densitometry analysis for the mitochondrial fusion protein Mfn1 was significantly reduced in the TH mice compared to the control mice (Mfn1 *p* = 0.0185), but the Mfn1 levels in the TH + chaya mice were significantly increased (Mfn1 *p* = 0.0195). No significant change in the level of Opa1 expression was detected in the TH mice compared to the control mice. * *p* = 0.01.

Mitochondrial dynamics proteins: As shown in Figure 3, the levels of mitochondrial fission proteins, Drp1 (*p* = 0.0325) and Fis1 (*p* = 0.0047), were elevated in the diabetic TH mice compared to the non-diabetic control mice, whereas, in the TH animals fed with chaya, the expression levels of Drp1 (*p* = 0.0024) were significantly lowered in the chaya-treated group. The expression levels of Fis1 also showed a decreasing trend in the chaya-treated mice relative to the untreated TH mice. The level of mitochondrial fusion protein Mfn1 (*p* = 0.0185) was lowered in TH mice, while the levels of Mfn1 (*p* = 0.0195) were significantly increased in chaya-fed TH mice, comparable to the levels in the non-diabetic control mice. However, the expression level of Opa1 did not show any significant change in the TH mice compared to the control mice. Further, there was no significant change in the expression level of this protein between the regular chow-fed and the chaya-fed TH mice. These results suggest that chaya treatment is capable of correcting the defect for mitochondrial dynamics proteins observed in the diabetic TH mice. * *p* = 0.01; ** *p* = 0.001; *** *p* = 0.0001.

**Figure 4:**
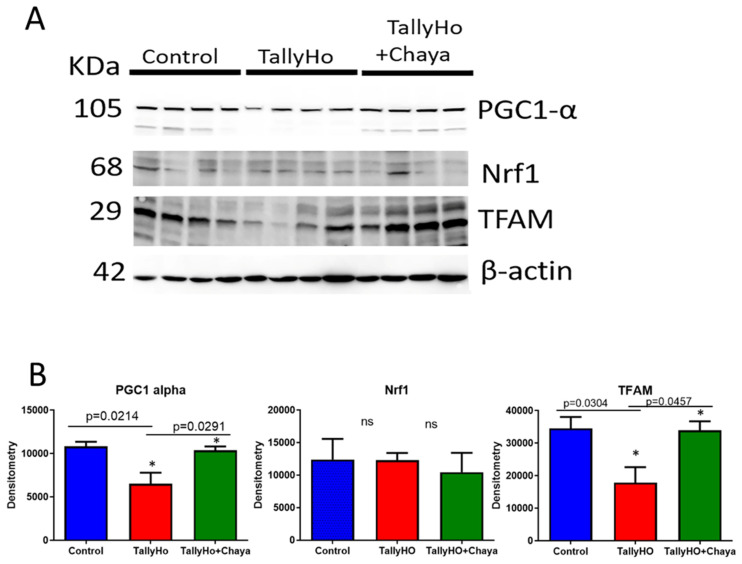
Immunoblotting analysis of mitochondrial biogenesis proteins using protein lysates obtained from the post-mortem brains of 14-month-old non-diabetic control mice, TH mice fed with chaya, and TH mice fed with regular chow (for each of the three groups, *n* = 4). (**A**) Immunoblots for the control, TH, and TH + chaya mice. (**B**) Quantitative densitometry analysis for the mitochondrial biogenesis proteins PGC1α and TFAM, which were significantly decreased in the TH mice compared to the control mice (PGC1α, *p* = 0.0214 and TFAM, *p =* 0.0304), but the PGC1α and TFAM levels were significantly increased in the TH + chaya mice at levels comparable to the control mice (PGC1α, *p* = 0.0291 and TFAM *p* = 0.0457). No significant change was seen in the expression level of Nrf1 in the TH mice compared to the control mice. * *p* = 0.01.

Mitochondrial Biogenesis Proteins.Figure 4 shows the levels of the mitochondrial biogenesis proteins in the TH mice and chaya-treated TH mice relative to the levels in the non-diabetic control mice. We see that the expression levels of PGC1α (*p* = 0.0214) and TFAM (*p* = 0.0304) were decreased in TH mice, while the expression of these proteins (PGC1α (*p =* 0.0291) and TFAM (*p* = 0.0457)) were increased in the chaya-treated TH mice group, similar to the levels of the non-diabetic control mice. The levels of Nrf1 did not show any significant change in the TH mice compared to the control mice.

**Figure 5:**
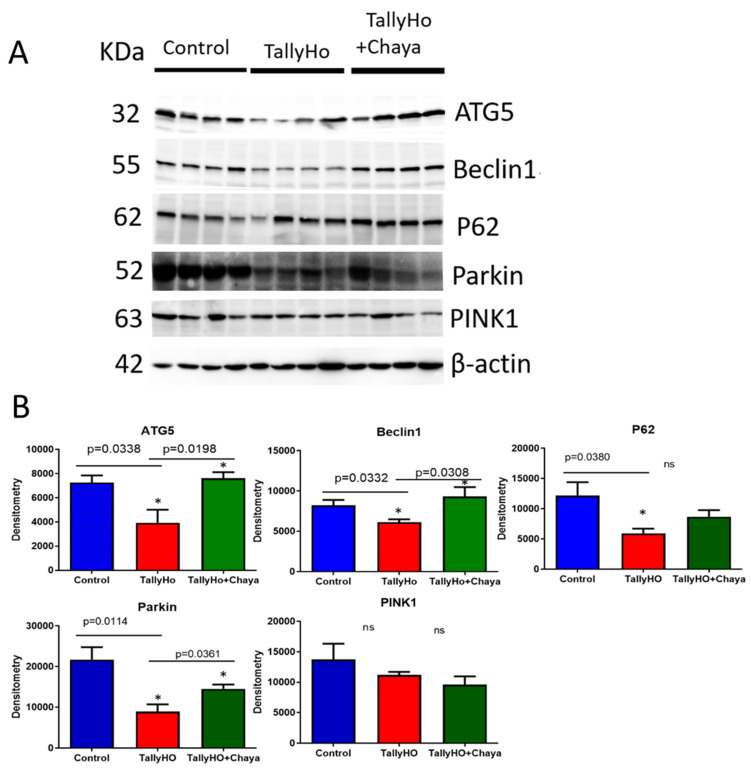
Immunoblotting analysis of autophagy and mitophagy proteins using protein lysates obtained from the post-mortem brains of 14-month-old non-diabetic control mice, TH mice fed with chaya, and TH mice fed with regular chow (for each of the three groups, *n* = 4). (**A**) Immunoblots for the control, TH, and TH + chaya mice. (**B**) Quantitative densitometry analysis for the autophagy and mitophagy proteins ATG5, Beclin1, P62, and Parkin, which were significantly decreased in the TH mice compared to the control mice (ATG5, *p* = 0.0338; Beclin1, *p* = 0.0332; P62, *p* = 0.0380; and Parkin, *p* = 0.0114). The ATG5, Beclin1, and Parkin levels were significantly increased in the TH + chaya mice at levels comparable to the control mice (ATG5, *p* = 0.0198; Beclin1, *p* = 0.0308; and Parkin, *p* = 0.0361). The levels of P62 in the TH + chaya mice showed an increasing trend, but the levels did not show statistical significance. PINK1 did not show any statistical significance between the TH mice and the control mice. * *p* = 0.01.

Autophagy/Mitophagy proteins.Figure 5 shows the protein expression of autophagy and mitophagy proteins. The levels of ATG5 (*p* = 0.0338), Beclin1 (*p* = 0.0332), P62 (*p =* 0.0380), Parkin (*p* = 0.0114), and PINK1 (*p* = 0.0445) were decreased in the TH mice compared to the non-diabetic control mice. However, the expression of these proteins (ATG5 (*p* = 0.0198), Beclin1 (*p* = 0.0308), and Parkin (*p* = 0.0361)) were increased in the chaya chow-fed TH mice, and the levels were comparable to the non-diabetic control mice. The expression level of the protein P62 in the chaya-treated TH mice compared to the untreated TH mice did not show statistical significance, but the level did show an increasing trend, similar to the non-diabetic control mice. The expression of PINK1 did not show any statistically significant change in TH mice compared to the non-diabetic control mice.

**Figure 6:**
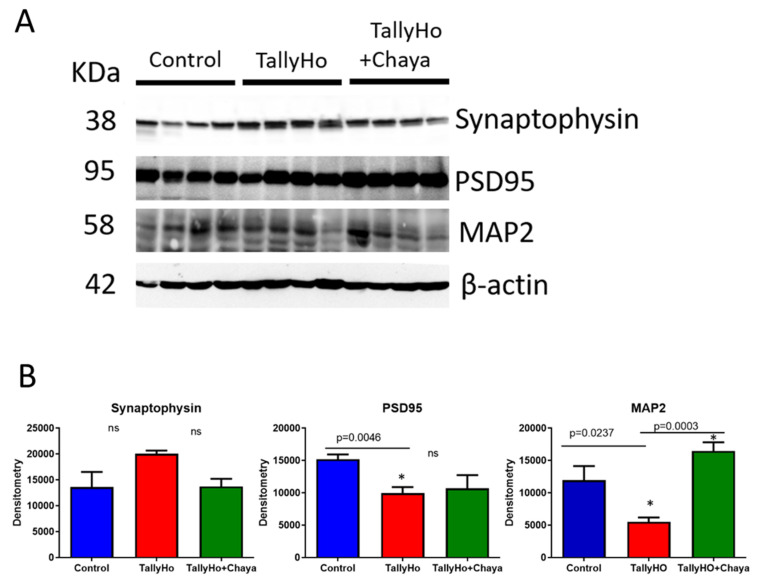
Immunoblotting analysis of mitochondrial synaptic and neuronal proteins using protein lysates obtained from the post-mortem brains of 14-month-old non-diabetic control mice, TH mice fed with chaya, and TH mice fed with regular chow (for each of the three groups, *n* = 4). (**A**) Immunoblots for the control, TH, and TH + chaya mice. (**B**) Quantitative densitometry analysis for the mitochondrial synaptic proteins synaptophysin, PSD95, and the neuronal protein MAP2. The expression levels of PSD95 and MAP2 were significantly decreased in the TH mice compared to the control mice (PSD95, *p* = 0.0046 and MAP2, *p* = 0.0237), but the levels of synaptophysin did not show any significant change in the TH mice compared to the control mice. MAP2 levels were significantly increased in TH + chaya mice at levels comparable to the control mice (MAP2, *p* = 0.0003). However, PSD95 did not show a significant change in the TH + chaya animals. * *p* = 0.01.

Synaptic and dendritic proteins. As shown in Figure 6, the expression levels of the synaptic protein PSD95 (*p* = 0.0046) and the dendritic protein MAP2 (*p* = 0.0237) were reduced in the TH mice compared to the control mice. While the expression level of MAP2 (*p* = 0.0003) was significantly increased in the chaya-fed TH mice compared to the regular chow-fed TH mice, the synaptic protein PSD95 did not show any significant change in the chaya-fed TH mice, and the synaptic protein synaptophysin did not show a significant change in the TH mice compared to the control mice.

### 3.4. Immunofluorescence Analysis

Immunofluorescence analysis was done to detect the localization of proteins, as described in Table 3, in the hippocampal sections of 14-month-old untreated TH mice, chaya-fed TH mice, and the age-matched non-diabetic control mice.

**Figure 7:**
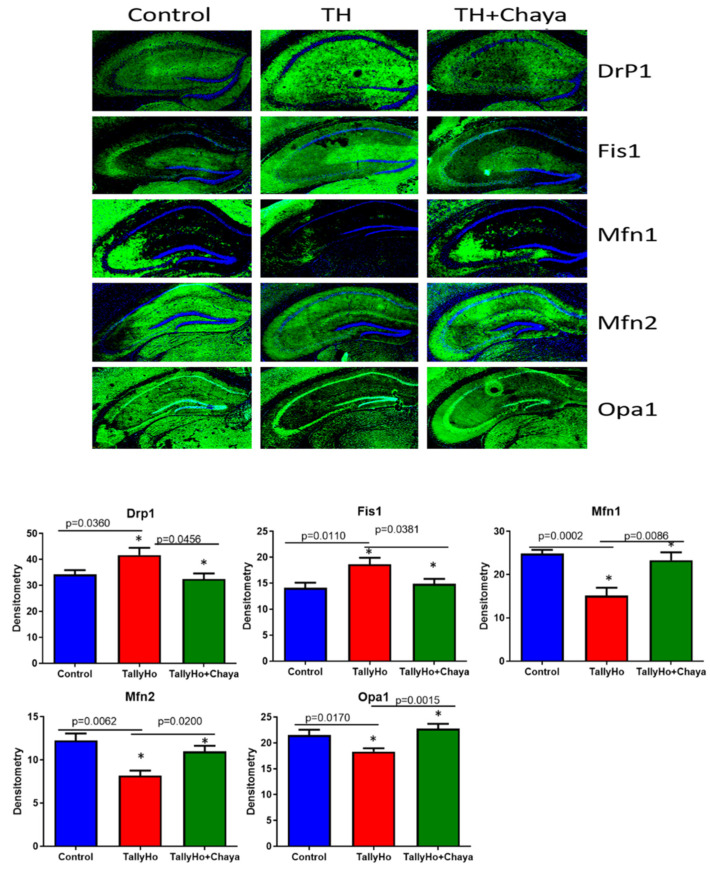
Immunofluorescence analysis of the hippocampal mitochondrial dynamic proteins of 14-month-old non-diabetic control mice, TH mice fed with chaya, and TH mice fed with regular chow. The TH + chaya mice showed significantly decreased levels of these proteins, which were increased in the TH mice (Drp1, *p =* 0.0456 and Fis1, *p* = 0.0381), and the TH + chaya mice showed significantly increased levels of these proteins, which were decreased in the TH mice (Mfn1, *p* = 0.0086; Mfn2, *p* = 0.0200; and Opa1, *p =* 0.0015). * *p* = 0.01.

Figure 7 shows the immunofluorescence analysis and the quantitative immunofluorescence analysis of the mitochondrial dynamic proteins. The mitochondrial fission proteins Drp1 (*p* = 0.0360) and Fis1 (*p* = 0.0110) showed significantly increased fluorescence in the regular chow-fed TH mice compared to the control mice. The expression levels of these proteins (Drp1 (*p* = 0.0456) and Fis1(*p* = 0.0381)) were significantly reduced in the chaya-fed TH mice compared to the regular chow-fed TH mice, similar to the control mice. The fluorescence in the mitochondrial fusion proteins Mfn1 (*p* = 0.0002), Mfn2 (*p* = 0.0062), and Opa1 (*p* = 0.0170) were significantly increased in the TH mice compared to the control mice. However, in the chaya-fed TH mice, the fluorescence seen for Mfn1 (*p* = 0.0086), Mfn2 (*p* = 0.0200), and Opa1 (*p* = 0.0015) were significantly reduced to levels comparable to those of the control mice.

**Figure 8:**
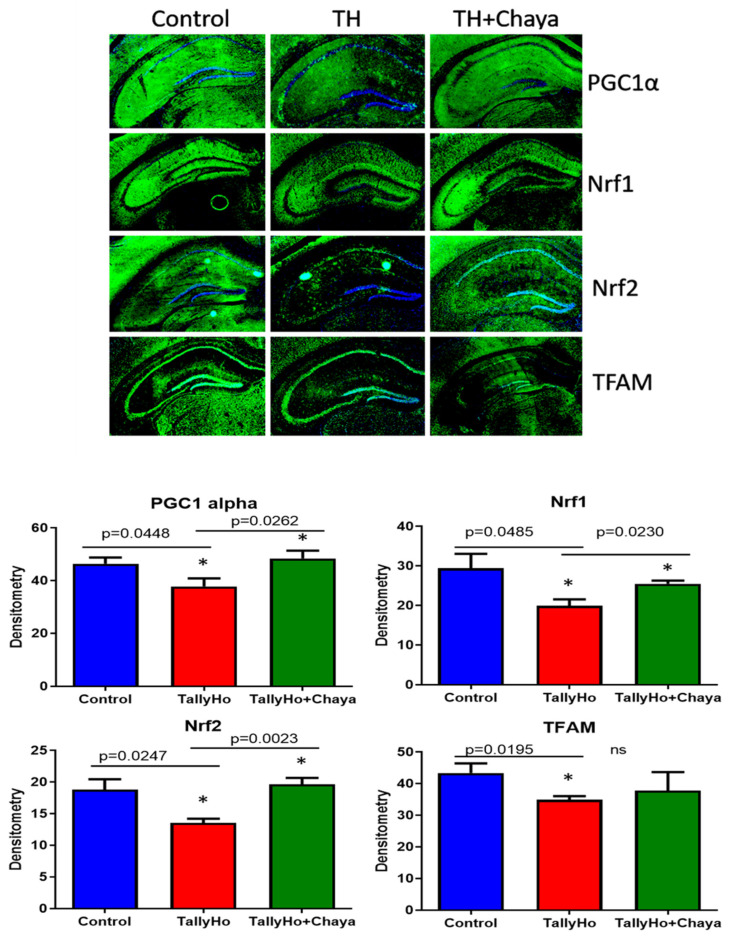
Immunofluorescence analysis of the hippocampal mitochondrial biogenesis proteins of 14-month-old non-diabetic control mice, TH mice fed with chaya, and TH mice fed with regular chow. The TH + chaya mice showed significantly increased levels of PGC1 alpha, Nrf1, and Nrf2, which were reduced in the TH mice (PGC1 alpha, *p* = 0.0262; Nrf1, *p* = 0.0230; and Nrf2, *p* = 0.0023). However, no significant changes were seen in the levels of the TFAM protein in the TH + chaya mice compared to the TH mice. * *p* = 0.01.

Figure 8 shows the immunofluorescence analysis and the quantitative immunofluorescence analysis of the mitochondrial biogenesis proteins. The fluorescence levels were significantly decreased in the hippocampal tissues stained with PGC1 alpha (*p =* 0.0448), Nrf1 (*p* = 0.0485), Nrf2 (*p* = 0.0247), and TFAM (*p* = 0.0195) in the TH mice compared to the control mice. In the chaya chow-fed TH animals, the fluorescence levels were significantly increased for PGC1 alpha (*p* = 0.0262), Nrf1 (*p* = 0.0230), and Nrf2 (*p* = 0.0023), which were at levels comparable to the control mice. However, the tissues stained with TFAM did not show any significant change with respect to the fluorescent intensity in the chaya chow-fed TH mice compared to the regular chow-fed TH mice.

**Figure 9:**
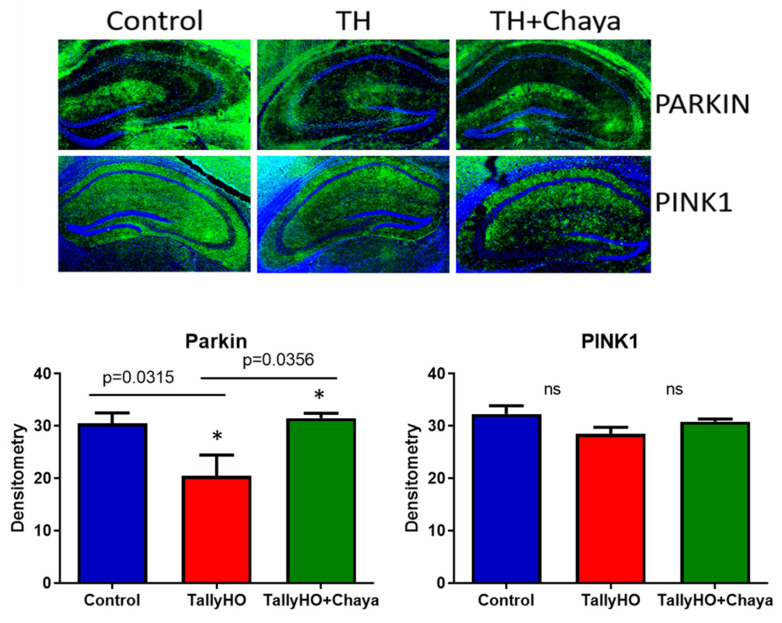
Immunofluorescence analysis of the hippocampal mitochondrial autophagy proteins Parkin and PINK1 of 14-month-old non-diabetic control mice, TH mice fed with chaya, and TH mice fed with regular chow. The TH + chaya mice showed significantly increased levels of Parkin, which was reduced in the TH mice (*p* = 0.0356). No significant changes were seen in the levels of PINK1 in TH mice compared to the control and in the TH + chaya chow fed mice compared to the TH mice. * *p* = 0.01.

Figure 9 shows the immunofluorescence analysis and the quantitative immunofluorescence analysis of the autophagy proteins Parkin and PINK1. While the levels of Parkin (*p* = 0.0315) were significantly decreased in the TH mice compared to the control mice, the levels of PINK1 did not show a significant change even though the fluorescent level of PINK1 appears to be decreased in the TH mice compared to the control mice. In the chaya chow-fed TH mice, the levels of Parkin (*p* = 0.0356) showed a significant increase compared to the regular chow-fed TH mice. The levels of PINK1 in the chaya chow-fed mice showed an increasing trend towards the control mice; however, the data obtained were not statistically significant for PINK1.

**Figure 10:**
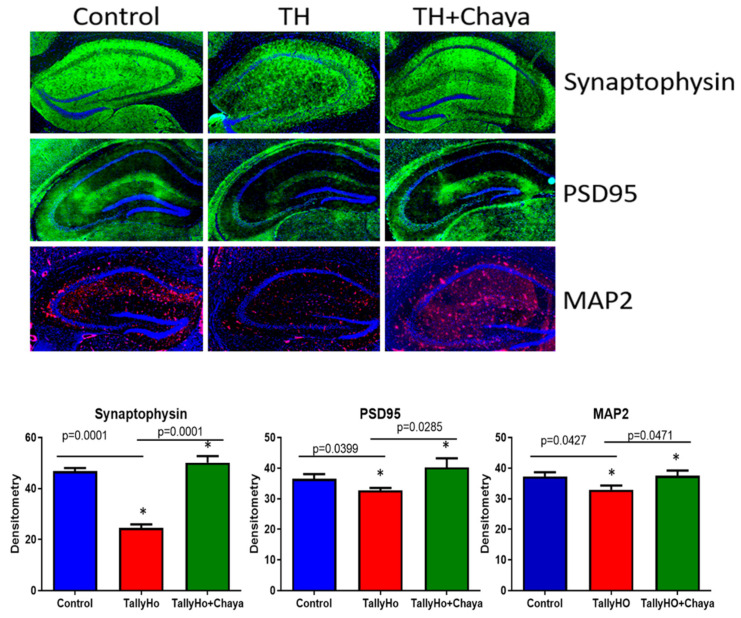
Immunofluorescence analysis of the hippocampal mitochondrial synaptic proteins and neuronal markers of 14-month-old non-diabetic control mice, TH mice fed with chaya, and TH mice fed with regular chow. The TH + chaya mice showed significantly increased levels of synaptophysin, PSD95, and Map2, which were reduced in the TH mice (synaptophysin, *p* = 0.0001; PSD95, *p* = 0.0285; and MAP2, *p =* 0.0471). * *p* = 0.01.

Figure 10 shows the immunofluorescence analysis and the quantitative immunofluorescence analysis of the synaptic proteins synaptophysin and PSD95 and the dendritic marker MAP2. Significantly decreased fluorescent levels of MAP2 (*p* = 0.0427), synaptophysin (*p* = 0.0001), and PSD95 (*p* = 0.0399) were seen in TH mice compared to non-diabetic control mice, and these levels were increased in the chaya chow-fed TH mice (MAP2 (*p* = 0.0471), synaptophysin (*p* = 0.0001), and PSD95 (*p* = 0.0285)) compared to the regular fed TH mice.

### 3.5. Mitochondrial Functional Assays

**Figure 11:**
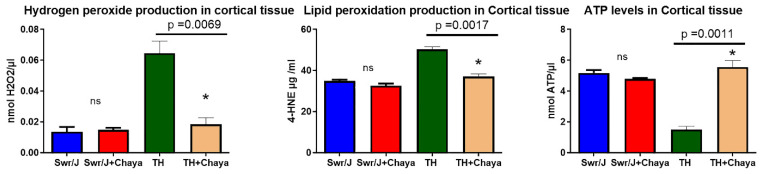
Mitochondrial functional parameters, including hydrogen peroxide, mitochondrial ATP, and lipid peroxidation (4-hydroxy-nonenol) were measured in the cortex of SWR/J control mice fed with regular chow and chaya chow, and TH mice fed with regular chow and chaya chow. The data are mean ± SD (*n* = 5 for each group). The TH + chaya mice showed significantly decreased levels of hydrogen peroxide (*p* = 0.0069) and lipid peroxidation (*p* = 0.0017), with significantly increased levels of ATP (*p* = 0.0011). * *p* = 0.01.

Figure 11 shows the results of the mitochondrial functional assays of the regular-fed and chaya-fed TH mice relative to their age-matched regular- and chaya-fed non-diabetic control mice.

The levels of hydrogen peroxide and lipid peroxidation (4-hydroxy-nonenol) were significantly increased in the TH mice compared to the non-diabetic control group. However, in the chaya-treated TH mice, the levels of hydrogen peroxide (*p* = 0.0069) and lipid peroxidation (*p* = 0.0017) were significantly reduced compared to the levels in the non-diabetic control mice.

The TH mice showed a significant reduction in ATP levels compared to non-diabetic control mice. In the TH mice treated with chaya, the ATP levels were significantly increased, similar to the non-diabetic control mice (*p* = 0.0011)

## 4. Discussion

The objective of our study was to determine the impact of the chaya leaf on mitochondrial and synaptic abnormalities in the Type 2 diabetes (T2D) mouse model TallyHo. In this study, we evaluated the experimental groups of TH diabetic mice fed with chaya chow and compared the results to a diabetic control group of TH diabetic mice fed with regular chow and a non-diabetic control group of SWR/J mice fed with regular chow. We assessed the body mass and fasting blood glucose in all the animals each week. After 11 weeks of study, we harvested the brain and other peripheral tissues. Using qRT-PCR and immunoblotting analyses, we measured mRNA abundance and the protein levels of mitochondrial biogenesis, mitochondrial dynamics, autophagy/mitophagy, and synaptic genes of the cerebral cortex. Using immunofluorescence analysis, we assessed the immunoreactivities of the mitochondrial and synaptic proteins in the hippocampus in order to understand if the changes in the cerebral cortex and hippocampus were similar.

We found increased body mass and blood glucose levels in the TH mice compared to non-diabetic control SWR/J mice. However, in the TH mice fed with chaya chow relative to the regular chow-fed TH mice, blood glucose levels and body mass were reduced to normal levels, indicating that chaya has protective properties against diabetes.

Our gene expression analysis showed increased levels of mitochondrial fission genes (Drp1 and fis1) and decreased levels of mitochondrial fusion genes (Mfn1, Mfn2, and Opa1) in TH mice relative to non-diabetic SWR/J control mice, indicating that mitochondrial dynamics were impaired in diabetic TH mice. Mitochondrial biogenesis, autophagy/mitophagy, and synaptic genes were reduced in TH mice relative to control mice. However, these were reversed in the chaya-chow fed TH mice. Overall, our findings suggest that chaya chow is protective against the diabetes-induced mitochondrial and synaptic mRNA levels in diabetic TH mice.

Our protein data agree with the mRNA levels, meaning the protein levels of mitochondrial biogenesis proteins, autophagy/mitophagy proteins, and the dendritic protein MAP2 were altered in the TH mice, but reversed in the chaya chow-fed TH mice relative to the regular chow-fed TH mice. Our immunofluorescence analysis revealed a similar pattern in the hippocampal tissues for all mitochondrial and synaptic proteins in non-diabetic SWR/J control mice and a reversal of these effects in the chaya chow-fed TH mice.

The effects of T2D on the brain’s structure and function are well studied. It has been identified that patients with T2D are at greater risk of developing AD than non-diabetic individuals. Hyperglycemia and hyperinsulinemia have been thought of as risk factors for cognitive decline and AD [26]. Normal mitochondrial biogenesis and dynamics are essential for correct synaptic function and transmission between neurons. Similarly, disruptions in autophagy/mitophagy processes also lead to mitochondrial dysfunction [27].

Our mitochondrial functional assays indicated a significant increase in the levels of hydrogen peroxide and lipid peroxidation in the TH mice and a significant reduction in the ATP levels in these mice compared to the non-diabetic control mice. However, in the chaya-treated TH mice, we observed that these levels were restored to the levels seen in the non-diabetic control mice.

These results suggest that hyperglycemia may play a key role in mitochondrial and synaptic functions in the brains of TH mice. This is in line with several other studies that have pointed towards brain changes linked with neurodegeneration and cognitive decline in diabetes [27,28]. More studies in relation to cognitive behavior patterns are definitely needed for TH mice.

Earlier studies in rodents showed that aqueous extracts of *Cnidoscolus* plant leaves had a strong potential for the treatment of insulin-dependent diabetes. These studies were conducted on the liver and pancreatic tissues of rodents to elucidate the effects of chaya extract on insulin secretion and pancreatic beta cells [10,11,12].

In this study, we have determined the protective effects of chaya on hyperglycemia-induced mitochondrial alterations in TH mice. In addition, since the long-term goal of our study is to understand the molecular links between Type 2 diabetes and late-onset Alzheimer’s disease, we have attempted to evaluate the mitochondrial and synaptic alterations in the brain tissues from TH mice fed with regular chow and the corresponding effects on chaya-fed TH mice. Our results strongly suggest that chaya is capable of reducing mitochondrial abnormalities and synaptic pathology in brains of TH male mice, besides reducing hyperglycemic conditions and obesity in TH mice. These findings are very significant to understanding the link between brain functions and diabetes. Our findings also indicate that chaya can be as natural diet supplement for treating prediabetic, diabetic subjects and also subjects with metabolic diseases.

Our future experiments will investigate the effect of chaya on other tissues, such as liver, pancreas, and kidney, in TH mice. We will also study the cognitive behavior pattern in regular chow- and chaya-fed TH mice with different age groups and the outcomes of these experiments will provide new insights into the development of AD in diabetic mice and explore a potential natural therapy for these age-related conditions.

## Figures and Tables

**Table 1:** Summary of qRT-PCR oligonucleotide primers used in measuring mRNA expressions in mitochondrial dynamics, mitochondrial biogenesis, synaptic, autophagy, and mitophagy genes in chaya chow-fed TH and regular chow-fed TH mice.

Gene	DNA Sequence (5-3)	PCR Product Size
	**Mitochondrial Dynamics Genes**	
Drp1	Forward Primer ATGCCAGCAAGTCCACAGAA	86
	Reverse Primer TGTTCTCGGGCAGACAGTTT	
Fis1	Forward Primer CAAAGAGGAACAGCGGGACT	95
	Reverse Primer ACAGCCCTCGCACATACTTT	
Mfn1	Forward Primer GCAGACAGCACATGGAGAGA	83
	Reverse Primer GATCCGATTCCGAGCTTCCG	
Mfn2	Forward Primer TGCACCGCCATATAGAGGAAG	78
	Reverse Primer TCTGCAGTGAACTGGCAATG	
Opa1	Forward Primer ACCTTGCCAGTTTAGCTCCC	82
	Reverse Primer TTGGGACCTGCAGTGAAGAA	
	**Mitochondrial Biogenesis Genes**	83
PGC1α	Forward Primer GCAGTCGCAACATGCTCAAG	
	Reverse Primer GGGAACCCTTGGGGTCATTT	
NRF1	Forward Primer AGAAACGGAAACGGCCTCAT	96
	Reverse Primer CATCCAACGTGGCTCTGAGT	
NRF2	Forward Primer ATGGAGCAAGTTTGGCAGGA	96
	Reverse Primer GCTGGGAACAGCGGTAGTAT	
TFAM	Forward Primer TCCACAGAACAGCTACCCAA	84
	Reverse primer CCACAGGGCTGCAATTTTCC	
	Reverse Primer AGACGGTTGTTGATTAGGCGT	
	**Autophagy/Mitophagy Genes**	
ATG5	Forward Primer TCCATCCAAGGATGCGGTTG	95
	Reverse Primer TCTGCATTTCGTTGATCACTTGAC	
PINK1	Forward Primer CCATCGGGATCTCAAGTCCG	70
	Reverse Primer GATCACTAGCCAGGGACAGC	
TERT	Forward Primer GCAAGGTGGTGTCTGCTAGT	100
	Reverse Primer AGCTTGCCGTATTTCCCCAA	
	**Synaptic Genes**	
Synaptophysin	Forward Primer CTGCGTTAAAGGGGGCACTA	81
	Reverse Primer ACAGCCACGGTGACAAAGAA	
PSD95	Forward Primer CTTCATCCTTGCTGGGGGTC	90
	Reverse Primer TTGCGGAGGTCAACACCATT	
	**Housekeeping Genes**	
β-actin	Forward Primer AGAAGCTGTGCTATGTTGCTCTA	91
	Reverse Primer TCAGGCAGCTCATAGCTCTTC	
GAPDH	Forward Primer TTCCCGTTCAGCTCTGGG	59
	Reverse Primer CCCTGCATCCACTGGTGC	

**Table 2:** Summary of the antibody dilutions and conditions used in the immunoblotting analysis of mitochondrial dynamics, mitochondrial biogenesis, synaptic, autophagy, and mitophagy proteins in the control, TH, and TH + chaya groups.

Markers	Primary Antibody (Species and Dilution)	Supplier	Secondary Antibody (Species and Dilution)	Purchased from Company, City, and State
β-Actin	Mouse monoclonal 1:1000	Sigma–Aldrich, St Luis, MO	Sheep Anti-mouse HRP 1:10,000	GE Healthcare Amersham, Piscataway, NJ
Drp1	Rabbit polyclonal 1:500	Novus Biological, Littleton, CO	Donkey Anti-rabbit HRP 1:10,000	GE Healthcare Amersham, Piscataway, NJ
Mfn1	Rabbit polyclonal 1:400	Novus Biological, Littleton, CO	Donkey Anti-rabbit HRP 1:10,000	GE Healthcare Amersham, Piscataway, NJ
Fis1	Rabbit polyclonal 1:500	Protein Tech Group, Inc., Chicago, IL	Donkey Anti-rabbit HRP 1:10,000	GE Healthcare Amersham, Piscataway, NJ
Opa1	Rabbit polyclonal 1:400	Novus Biological, Littleton, CO	Donkey Anti-rabbit HRP 1:10,000	GE Healthcare Amersham, Piscataway, NJ
Nrf1	Rabbit polyclonal 1:400	Cell Signalling Technology, Danvers, MA	Donkey Anti-rabbit HRP 1:10,000	GE Healthcare Amersham, Piscataway, NJ
MAP2	Rabbit monoclonal 1:600	Invitrogen, Waltham, MA	Donkey Anti-rabbit HRP 1:10,000	GE Healthcare Amersham, Piscataway, NJ
ATG5	Rabbit polyclonal 1:400	Novus Biological, Littleton, CO	Donkey Anti-rabbit HRP 1:10,000	GE Healthcare Amersham, Piscataway, NJ
Beclin1	Rabbit polyclonal 1:400	Novus Biological, Littleton, CO	Donkey Anti-rabbit HRP 1:10,000	GE Healthcare Amersham, Piscataway, NJ
TFAM	Rabbit polyclonal 1:400	Novus Biological, Littleton, CO	Donkey Anti-rabbit HRP 1:10,000	GE Healthcare Amersham, Piscataway, NJ
PGC 1α	Rabbit polyclonal 1:3000	Novus Biological, Littleton, CO	Donkey Anti-rabbit HRP 1:10,000	GE Healthcare Amersham, Piscataway, NJ
Synapto physin	Rabbit polyclonal 1:3000	Novus Biological, Littleton, CO	Donkey Anti-rabbit HRP 1:10,000	GE Healthcare Amersham, Piscataway, NJ
PSD95	Rabbit polyclonal 1:400	Cell Signalling Technology, Danvers, MA	Donkey Anti-rabbit HRP 1:10,000	GE Healthcare Amersham, Piscataway, NJ
PARKIN	Mouse monoclonal 1:600	Novus Biological, Littleton, CO	Donkey Anti-rabbit HRP 1:10,000	GE Healthcare Amersham, Piscataway, NJ
PINK1	Rabbit polyclonal 1:400	Novus Biological, Littleton, CO	Donkey Anti-rabbit HRP 1:10,000	GE Healthcare Amersham, Piscataway, NJ
SQSTM1/P62	Rabbit polyclonal 1:1000	Cell Signalling Technology, Danvers, MA	Donkey Anti-rabbit HRP 1:10,000	GE Healthcare Amersham, Piscataway, NJ

**Table 3:** Summary of the antibody dilutions and conditions used in the immunofluorescence analysis of the mitochondrial dynamics, mitochondrial biogenesis, synaptic, autophagy, and mitophagy proteins in the brain sections of non-diabetic mice, chaya-fed mice, and regular chow-fed diabetic TH mice.

Markers	Primary Antibody (Species and Dilution)	Supplier	Secondary Antibody (Species and Dilution)	Purchased from Company, City, and State
Drp1	Rabbit polyclonal 1:100	Novus Biological, Littleton, CO	Donkey anti-rabbit IgG Alexa Fluor 488	Thermo Fisher Scientific, Waltham, MA
Fis1	Rabbit polyclonal 1:100	Protein Tech Group, Inc., Chicago, IL	Donkey anti-rabbit IgG Alexa Fluor 488	Thermo Fisher Scientific, Waltham, MA
Opa1	Rabbit polyclonal 1:100	Novus Biological, Littleton, CO	Donkey anti-rabbit IgG Alexa Fluor 488	Thermo Fisher Scientific, Waltham, MA
Mfn1	Rabbit polyclonal 1:100	Novus Biological, Littleton, CO	Donkey anti-rabbit IgG Alexa Fluor 488	Thermo Fisher Scientific, Waltham, MA
Mfn 2	Rabbit polyclonal 1:100	Novus Biological, Littleton, CO	Donkey anti-rabbit IgG Alexa Fluor 488	Thermo Fisher Scientific, Waltham, MA
PGC 1α	Rabbit polyclonal 1:100	Novus Biological, Littleton, CO	Donkey anti-rabbit IgG Alexa Fluor 488	Thermo Fisher Scientific, Waltham, MA
Nrf1	Rabbit polyclonal 1:100	Cell Signalling Technology, Danvers, MA	Donkey anti-rabbit IgG Alexa Fluor 488	Thermo Fisher Scientific, Waltham, MA
Nrf2	Rabbit polyclonal 1:100	Cell Signalling Technology, Danvers, MA	Donkey anti-rabbit IgG Alexa Fluor 488	Thermo Fisher Scientific, Waltham, MA
TFAM	Rabbit polyclonal 1:100	Novus Biological, Littleton, CO	Donkey anti-rabbit IgG Alexa Fluor 488	Thermo Fisher Scientific, Waltham, MA
PARKIN	Mouse monoclonal 1:100	Novus Biological, Littleton, CO	Donkey anti-rabbit IgG Alexa Fluor 488	Thermo Fisher Scientific, Waltham, MA
PINK1	Rabbit polyclonal 1:100	Novus Biological, Littleton, CO	Donkey anti-rabbit IgG Alexa Fluor 488	Thermo Fisher Scientific, Waltham, MA
Synapto physin	Rabbit polyclonal 1:100	Novus Biological, Littleton, CO	Donkey anti-rabbit IgG Alexa Fluor 488	Thermo Fisher Scientific, Waltham, MA
PSD95	Rabbit polyclonal 1:100	Cell Signalling Technology, Danvers, MA	Donkey anti-rabbit IgG Alexa Fluor 488	Thermo Fisher Scientific, Waltham, MA
MAP2	Rabbit polyclonal 1:100	Invitrogen, Waltham, MA	Goat anti-mouse IgG Alexa Fluor 594	Thermo Fisher Scientific, Waltham, MA

**Table 4:** A comparison of the mRNA fold changes of mitochondrial structural, synaptic, biogenesis, autophagy, and mitophagy genes in TH mice fed with chaya and TH mice fed with regular chow.

Biological Pathways	Genes	mRNA Fold Change TallyHO vs. Control	mRNA Fold Change Chaya vs. TallyHO
Mitochondrial structure	DRP1	5.7	−0.78
	FIS1	6.7	−0.75
	MFN1	−4.1	2.6
	MFN2	−4.5	2.4
	OPA1	−3.1	1.1
Biogenesis	NRF1	−0.5	0.2
	NRF2	−0.6	0.4
	TFAM	−1.1	1.98
	PGC1-α	−2.0	0.97
Autophagy	ATG5	−3.1	3.9
	PINK1	−4.6	8.7
Mitophagy	TERT	−4.1	3.4
Synaptic	Synaptophysin	−4.0	2.5
	PSD95	−3.7	3.4

## Data Availability

Not applicable.
